# Source Analysis of Heavy Metal Pollution Using UNMIX and PMF Models in Soils along the Shuimo River in Urumqi, China

**DOI:** 10.3390/ijerph192214794

**Published:** 2022-11-10

**Authors:** Honggang Zang, Yidan Zhang, Junqin Yao, Huiying Ma

**Affiliations:** 1 Department of Environment, College of Ecology and Environment, Xinjiang University, Urumqi 830017, China; 2 Key Laboratory of Osis Ecological Education, Urumqi 830017, China

**Keywords:** PMF, UNMIX, source analysis of heavy metals, soil along the river

## Abstract

Eight kinds of heavy metals in soil within 0–2 km from the banks of Shuimo River in Urumqi were analyzed by using an X-ray fluorescence spectrometer and national standard detection methods. Unmix and PMF models are comprehensively used to analyze potential pollutant sources and contribution rates. Soil samples are sampled in three layers of 0–20, 20–40, and 40–60 cm, and each group of sample points in each layer is 5 m, 1 km, and 2 km away from the riverbank, respectively. Only the average concentration of Mn in each layer of soil is lower than the background value, according to the analytical results, while the average concentration of other heavy metals surpasses the background value. The highest proportion of exceeding the background value is Ni in the 40–60 cm soil layer, up to 1.92 times. Unmix and PMF models are used to analyze pollutants’ source quantity and contribution rate, respectively. The results show that the two models can identify two pollution sources at the three soil layers, and their contribution rates are similar, and each index of the analysis results of the two models is within the required range of model reliability. By comparing with the Pearson correlation coefficient and distribution map of heavy metal concentration in surface soil, it is concluded that Zn, Pb, Cr, and Cu are mainly from industrial sewage and air pollution from coal combustion, while As, Mn, Ni, and V are mainly from agricultural pollution and light industrial pollution. In future research, it is necessary to investigate the change of heavy metal concentration in detail from the time dimension to further quantitatively calculate the potential pollutant source and contribution rate.

## 1. Introduction

Soil heavy metal pollution is an important issue in environmental science, and heavy metal pollution in urban soil has received increasing research attention [1,2,3]. With industrial development, human activities have become intense, leading to high contents and diversity of heavy metals in urban soils. Being toxic and harmful pollutants that are difficult to degrade, heavy metals not only cause soil quality degradation and reduce crop yield and quality but also affect human health through food chain accumulation, dust inhalation, skin contact, and other ways and induce fatal diseases, such as cancer [4]. Moreover, treating soil pollution is more difficult and time-consuming than treating air and water pollution [5]. After entering the soil, heavy metals exhibit characteristics such as concealment, hysteresis, accumulation, and irreversibility [6], thereby directly or indirectly endangering human life and health through the food chain [7,8,9].

Soil is important for agricultural production and human survival [10]. Heavy metal pollution in China is not improving, and strict actions must be undertaken to control heavy metal pollution and reduce its accumulation risk [11,12]. Pollution source allocation is a key step in addressing pollution threats. Existing heavy metal source analysis methods can be divided into two major categories: qualitative pollution source identification and quantitative pollution source analysis [13]. The former primarily determines the pollution source via geostatistical and multivariate statistical analyses. It can identify possible pollution sources of heavy metals and their major pollution points [14,15,16,17]. At present, the main models used to calculate the contribution of pollution sources to pollutants are multivariate linear regression after absolute principal component analysis (APCs-MLR), chemical mass balance (CMB) model, positive matrix factorization (PMF), and UNMIX [18,19,20,21]. Among them, the PMF and UNMIX models approved by the U.S. Environmental Protection Agency can not only provide the number of pollution sources and the elements contributed by each pollution source but also provide the contribution rate of each pollution source to the elements [22,23,24]. Although they have been widely used in the source analysis of air, water, and sediment pollutants [25,26,27,28], they are rarely used in analyzing soil heavy metal pollution sources [29]. Previous studies have conducted source analysis of heavy metal pollution in the soil along the Shuimo River [30]. Using an analysis method that combines UNMIX and PMF is recommended for source analysis. Thus, by combining the two models, we can overcome both models’ shortcomings and potential errors and obtain more reliable results.

In addition, it is an effective “point to surface” method to use geostatistical analysis and geographic information systems to interpolate the pollutant concentration of soil sample points, analyze the spatial distribution characteristics of soil heavy metal pollution, and mine the spatial distribution rules. The ordinary Kriging interpolation method is an advanced statistical process of generating estimated surfaces through a group of scattered points with z values. Compared with the inverse distance weighted interpolation method, the ordinary Kriging interpolation method takes into account the spatial correlation problem [31] and solves the problem that it is difficult to estimate the error compared with the spline interpolation method [32]. When there are many data points, ordinary Kriging’s spatial interpolation method is more reliable and often used in the field of soil science [33]. Therefore, this paper uses the ordinary Kriging method and PMF and UNMIX receptor models to explain the source and distribution of heavy metal pollution in space, so that the distribution of pollution sources is more intuitive.

## 2. Geography of the Shuimo River

Urumqi is the capital of the Xinjiang Uyghur Autonomous Region. It is located on the northern slope of the Tianshan Mountains and is surrounded by desert. It is located between 42°45′32.4″–44°08′00″ N and 86°37’33.3″–88°58’24.4″ E. Urumqi is located in the center of the Eurasian Continent, Northwest China, and central Xinjiang. It is the bridgehead of the Second Eurasian Continental Bridge in western China and an important gateway for China to open to the west. Urumqi is approximately 153 km long from north to south and 190 km wide from east to west, with a total area of 12,000 km^2^.

Shuimo River originates from Abuja Hasmudara in the southwest of Fukang City, Changji Hui Autonomous Prefecture. The river is mainly located in the eastern suburb of Urumqi. It flows into Bayi Reservoir through Shuimogou District, Tianshan District, Midong District of Urumqi, and finally flows into Bayi Reservoir. It is a rock fissure spring river mainly recharged by groundwater. SShimoRiver basin covers E 87°59′~E88°09′, N 43°45′~N 44°15′, with a drainage area of 281.4 km^2^, a water source protection area of 45.7 km^2^ and a total length of 50 km. The southeast part of the basin is medium-low mountains, the middle part is low mountains and hills, and the north gradually becomes a plain area, with an altitude of 600~1800 m [34]. The surface water resource of Shuimo River is 21.6089 million m^3^/year, and the groundwater resource is 34.4759 million m3/year. The annual runoff is relatively stable, without an obvious dry season. The average annual runoff is 39.67 million m^3^/year (Weihuliang Observation Station) [35].

## 3. Sample Collection and Analysis

### 3.1. Sample Collection

**Figure 1:**
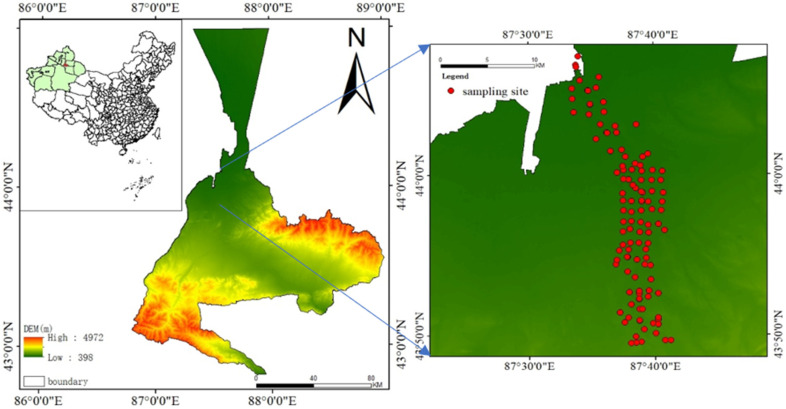
Distribution of sampling points.

In total, 42 soil sample points were placed along the Shuimo River bank using the grid method (2 km × 2 km). In addition, parallel sample points were set at 1 and 2 km along the vertical direction of the riverbank of each soil sample point, and 80 parallel soil sample points were thus collected. For each sample point, three layers of soil samples at depths of 0–20, 20–40, and 40–60 cm were collected vertically from the ground surface at every 20 cm depth [30,36]. Some of the sampling sites were too stiff for the soil texture and therefore only 0–20 cm or two soil layers of 0–20 and 20–40 cm, and approximately 1 kg of soil samples was collected for each layer of soil. A total of 122 soil sample sites were collected along the Shuimo River bank, and the number of soil samples was 334. When collecting sediment samples, try to choose a place with gentle water flow, and use the plum blossom mixed sampling method to randomly take 5 times sediments with a depth of 0~10 cm to mix and record them as a sediment sample. A total of 23 surface sediment samples at the bottom of the river were collected along the river channel, and they were sealed and brought back at 4 °C. Figure 1 shows the distribution of the sampling points. The coordinate system used in the drawing is WGS 1984 in the geographical coordinate system.

### 3.2. Sample Analysis

Gravel and plant residues were removed from the soil samples, dried in natural shade indoors, grounded, and passed through 10-mesh and 100-mesh nylon screens to determine soil pH and heavy metal content, respectively. A portable X-ray fluorescence spectrum analyzer (Thermo Scientific Nixon XL3t GOLDD analyzer, Thermo Fisher, Wortham, Massachusetts, USA) was used to rapidly identify all samples to be tested. The test was set to the soil mode of 90 s, repeated thrice, and the average value was obtained. Simultaneously, 30% of the samples were tested via the national standard method to calibrate the data measured by the X-ray fluorescence spectrum analyzer and ensure its accuracy. The national standard method test was divided into two stages: pretreatment and determination. First, an acid solution method was used for the pretreatment. We accurately weighed 0.5000 g of soil sample into a polytetrafluoroethylene crucible, wetted it with 2–3 drops of high-purity water, and filled it in the order of HF → HNO_3_ → HClO_4_ in strict accordance with the dosage. We removed the boiled sample for cooling when it emitted slight white smoke. Subsequently, we added HF → HClO_4_ to digest to paste, and the paste residue was dissolved in HNO_3_ at low temperature and washed into the colorimetric tube [37]. After pretreatment, the arsenic content (As) was determined via microwave digestion/atomic fluorescence spectrometry (Aurora lumina 3400 atomic fluorescence spectrometer, Aurora Instruments, Vancouver, British Columbia, CAN ), and the contents of nine heavy metals (Co, Zn, Cu, Ni, and Cr) were determined via flame atomic absorption spectrometry (PerkinElmer aa900 atomic absorption spectrometer, PerkinElmer, Waltham, MA, USA). Compared to the national standard method, this method has an accuracy of over 95%. The above-mentioned results show that the test results of the X-ray fluorescence spectrum analyzer are more accurate and convenient. Therefore, all data measured by this instrument were used for statistical analysis in this study to reduce the error.

### 3.3. Quality Assurance and Control

The testing standards refer to HJ/T166-2004 Technical Code for Soil Environmental Monitoring and Modern Analysis Methods of Soil Elements of China National Environmental Monitoring Station. Compared with the national standard method, the accuracy of the fast-testing method used is more than 95%.

## 4. Introduction to Models

### 4.1. PMF Model

Paatero and Tapper proposed the concept of the PMF model in 1994. It is a source analysis method based on factor analysis and has been updated to PMF version 5.0. When a dataset with n samples and m chemical species is imported into the PMF model, it is considered that the dimension of Matrix *X* is n × m [38]. This model decomposes the original matrix *X_ij_* into two-factor matrices, *g_ik_* and *f_ik_*, and residual matrix *e_ij_*. The formula used is as follows:

(1) $$X_{ij}=\sum_{k=1}^{p}g_{ik}f_{ik}+e_{ij}$$

where *X_ij_* is the concentration of the *j*-th heavy metal in sample *i*, *g_ik_* is the contribution rate of the *k*-th pollution source in sample *i*, and *f_ik_* is the characteristic value of the concentration of *k*-th heavy metal of pollution source *i*. The residual matrix *e_ij_* was calculated from the minimum value of the objective function Q.

(2) $$Q=\sum_{i=1}^{n}\sum_{j=1}^{m}{\left(\frac{e_{ij}}{u_{ij}}\right)}^{2}$$

where *u_ij_* is the uncertainty of the *j*-th heavy metal in sample *i*. The MDL calculation method followed that described by Chen et al. [39].

### 4.2. UNMIX Model

In 2003, Henry proposed a calculation method for UNMIX model and launched the UNMIX model software on the official website of the EPA of the United States. The UNMIX model provides a visible graphical interface, and graphical diagnostic tools can be included in the results. The model obtains the results according to the selected components and expresses the uncertainty information in the analysis results, which partly reduces the impact of anthropogenic factors [40]. The UNMIX model has a strict data screening process based on eigenvalue analysis, thereby eliminating missing data or data lower than the minimum detection limit. As this model uses the geometric concept of self-modeling curve resolution, the model results must be non-negative. Using the singular value decomposition (SVD) method, the model estimates the number of sources by reducing the dimension of the data space m to *p* [29,41]. The calculation formula is as follows:

(3) $$C_{ij}=\sum_{k=1}^{m}F_{jk}S_{jk}\;+E$$

where *C_ij_* is the content of the *j*-th element in the i-th regional sample (soil); *F_jk_* is the element content of the *j*-th sample in source *k* (*k* = 1, … *M*), representing the composition of the source; *S_jk_* represents the total amount of source *K* in the *i*-th sample, that is, the contribution rate of the source; and E is the uncertainty of the analysis process or the standard deviation of each source component. In this study, to eliminate the influence of the huge difference between different trace heavy metals in the soil on the analysis results, before inputting the data into the UNMIX model, the deviation normalization method was used to process the whole data. The calculation formula is as follows:

(4) $$X_{k}=\frac{X_{i}-MinX_{i}}{MaxX_{i}-MinX_{i}}$$

Deviation standardization was applied to 2856 data points from 357 soil sample sites containing eight heavy metals (Pb, Zn, Cu, Ni, Mn, Cr, As, and V). The normalized data were dimensionless, and the value of the variable observations ranged between 0 and 1. Standardized data were replaced with UNMIX 6.0 for analysis.

## 5. Results and Discussion

### 5.1. Analysis of Heavy Metal Pollution along the Shuimo River

According to the soil layers, the collected soil samples were divided into three parts. Since some places have high walls, private houses, and steep slopes, samples of individual soil layers were not collected, and therefore, the number of samples of each soil layer was inconsistent. There were 121 samples in the 0–20 cm soil layer, 109 samples in the 20–40 cm soil layer, 104 samples in the 40–60 cm soil layer, and 23 samples in the sediment soil layer. Since the number of sediment soil layer samples was small, and the statistical significance was not significant, statistical characteristics and correlation analysis were not conducted.

Based on the statistical results of heavy metal contents in the soil along the Shuimo River (Table 1), the average concentration values of As, Pb, Zn, Cu, Ni, Mn, Cr, and V were 12.65, 20.00, 80.84, 38.78, 49.70, 532.47, 53.18, and 111.29 mg/kg, respectively. Moreover, the average concentrations of all heavy metals except Mn were higher than the background values of the corresponding local soil heavy metals, and the average concentration of Ni in each layer highly exceeded the background value concentration of Xinjiang Uygur Autonomous Region in the *China Soil Element Background Value* issued by the National Environmental Protection Administration in 1990, reaching 1.85, 1.87 and 1.92 times, respectively. Based on the degree of coefficient of variation [42], Zn, Pb, As, Cr, and Cu was highly variable (CV > 30%), indicating that the spatial distribution of these five heavy metals varies, and the source may be significantly affected by external interference. Ni, Mn, and V were moderately variable (10% < CV < 30%), indicating that these three heavy metals are relatively evenly distributed. The concentrations of all heavy metals in this study were lower than the screening value of the GB 36600-2018 soil environmental quality control standard for soil pollution risk of construction land (Trial) (Zn, Mn, and V have no screening value, and Cr screening value is hexavalent), indicating that the soil environmental quality along the Shuimo River is generally good. However, compared with the background value, the eight heavy metals were enriched and may pose a pollution risk, and thus, further pollution source analysis is warranted.

### 5.2. Correlation Analysis of Heavy Metal Contents

**Figure 2:**
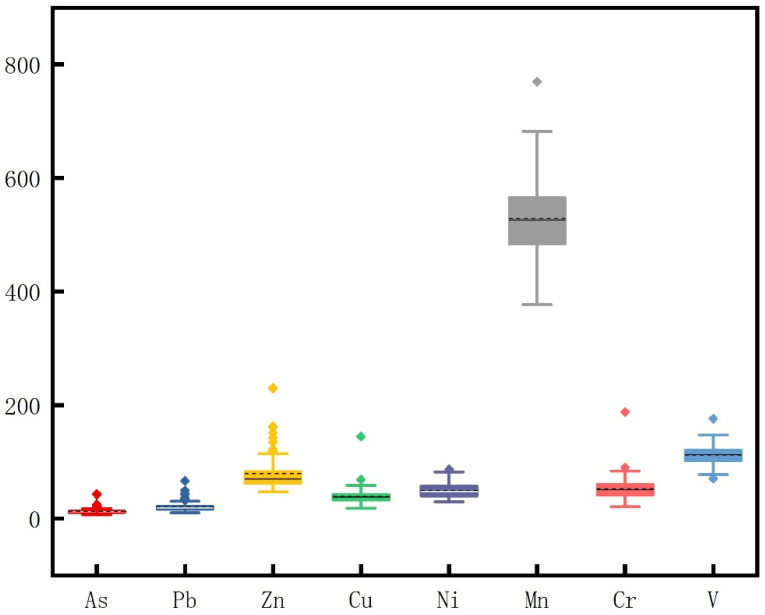
Box Diagram of Eight Heavy Metals Concentrations.

The correlation between heavy metal contents can provide information about heavy metal sources. As seen in Figure 2, Pearson was adopted to describe the correlation since the set of data followed a normal distribution.

Table 2 presents the Pearson correlation coefficient analysis results for the heavy metal content in soils along the Shuimo River. As presented in Table 2, Zn and Pb were significantly positively correlated in all soil layers (*p* < 0.01), and Cu was significantly positively correlated with these two metals in all soil layers (*p* < 0.05), indicating that these three elements may have similar sources. As and Cu and As and V also had a certain correlation in each soil layer (*p* < 0.05); however, no significant correlation was observed between Cu and V, and there was no correlation between Ni and other heavy metals. Pearson’s correlation coefficient can only be used as a reference for heavy metal source analyses, and thus, it cannot be used for reaching conclusions. Further verification and analysis are required to accurately determine the source of heavy metals.

### 5.3. Source Analysis of UNMIX and PMF Models

**Figure 3:**
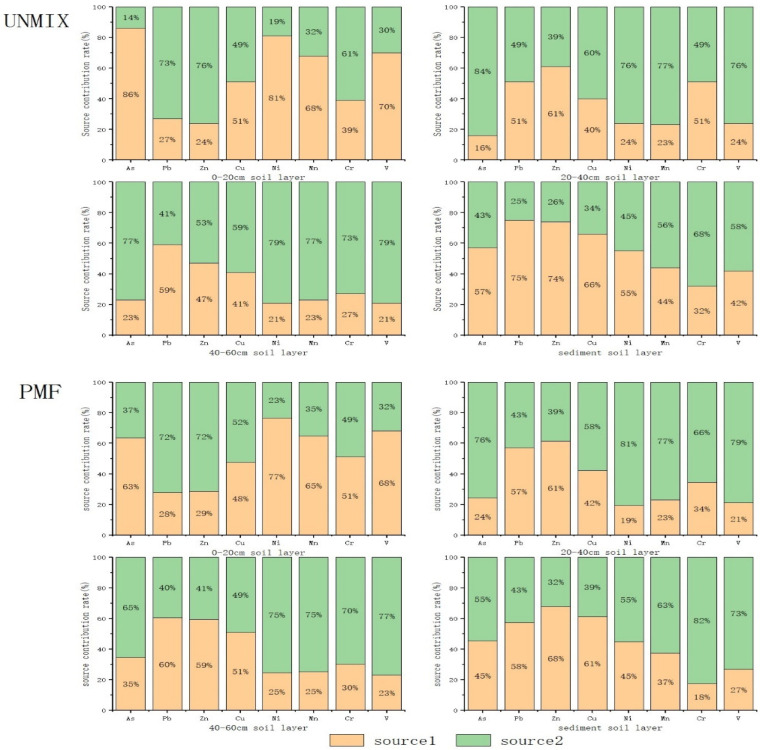
Contribution rates of different heavy metal sources in the study area (UNMIX and PMF).

The samples of four soil layers were analyzed using the UNMIX and PMF models, respectively. After importing the data into the UNMIX software, use the Suggest exclusion function to screen the data, and the results show that all eight substances meet the software operation requirements. After the official operation, the software automatically matches the two sources and fits the proportion of each source. The first step is to import the data and the uncertainty corresponding to the data into the PMF software. Next, set the number of the run to 20 and the number of factors to 2. Select the operation result with the largest Q (Robust) value to record the proportion of each source. After sorting out the operation results of the two models, Figure 3 is drawn with Origin software. As shown in the figure, both the UNMIX and PMF models determined the two sources of heavy metals in the soil. For the UNMIX model, min RSQ is the minimum r^2^ of any species in the model (the r^2^ of any species is greater than this value). Our results show that the minimum RSQ was 0.91, indicating that 91% of the species variance can be explained by the model, which is larger than the minimum value required by the model (min RSQ > 0.8); Min sig/noise = 3.05 in multiple operations, which was higher than the minimum value required by the system (min sig/noise > 2). For PMF, the minimum R^2^ in the three-layer soil operation was between 0.233 and 1, Q(robust)/Q (true) tended to converge, and the residual error was between −3 and 3, indicating that the model results can efficiently explain the source of soil heavy metals [43]. Therefore, the analysis results of these two models were reliable. As shown in Figure 3, according to the UNMIX and PMF analysis results, the proportions of Zn and Pb sources in most soil layers are approximately the same, indicating that these two heavy metals likely have the same source. The Pearson correlation coefficient analysis results presented in Table 2 also reveal that Pb and Zn are significantly correlated, which further supports this view. By comparing the source proportions of each soil layer, the source proportions of different soil layers were significantly different, indicating that different pollution sources have different effects on different soil layers. When the Pb and Zn sources increase (decrease), other heavy metals decrease (increase), indicating that other heavy metals may have different Pb and Zn sources. The analysis results of the two models for the three soil layers of 0–20 cm, 20–40 cm, and 40–60 cm were very similar; however, the difference in the sediment layer was relatively high. The sediment layer is considered to have very few samples, resulting in large fluctuations in the model analysis results; thus, the reliability is low. According to the analysis results of the two models, the Source 1 contributions of As, Mn, Ni, and V were primarily concentrated in the surface soil, and the analysis results of the UNMIX model were more obvious than those of the PMF model, indicating that these four heavy metals may belong to the same source and tend to be caused by later human activities.

### 5.4. Surface Distribution of Heavy Metal Concentration via the Kriging Interpolation Method

**Figure 4:**
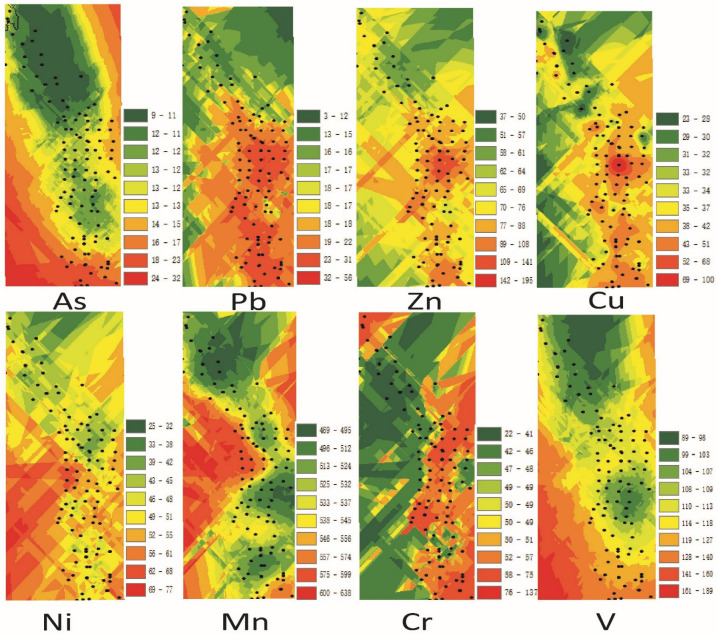
Distribution of heavy metal concentration in 0–20 cm soil layer.

Only the surface soil was sampled completely among the three soil layers, and other soil layers could not be excavated entirely because of various factors; therefore, the sampling points were incomplete. Since the expected value of attribute value is assumed to be unknown, the ordinary Kriging interpolation method is used to draw the spatial distribution map of pollutants. As shown in Figure 4, we used the ordinary Kriging interpolation method to draw the distribution map of heavy metal concentration in the 0–20 cm soil layer and further analyzed the heavy metal sources using this map. Based on the correlation analysis, it can be seen that there is a significant correlation among the three heavy metals Zn, Pb, and Cu, and it can be seen from the comparison of different soil layers in Figure 2 that the change rule of source percentage among As, Mn and V is almost the same. According to the distribution law of heavy metal concentration in Figure 4, it can be found that the concentration of Zn, Pb, Cr, and Cu in the south end of the river is significantly higher than that in the north end. This shows that the four heavy metals Zn, Pb, Cr, and Cu come from one of the pollution sources, and the pollution source is concentrated in the south end of the river. According to the survey and relevant documents, Weihuliang Coal Mine, Weihuliang Power Plant, Cement Plant, Yinjia Paper, and other enterprises and many domestic waste storage sites are distributed within two kilometers around the sampling point at the south end. Studies have found that Cu and Zn mainly come from garbage incineration and industrial emissions, and some also come from coal emissions [44]. It is generally believed that Pb mainly comes from coal combustion, industrial utilization of lead-containing ores, combustion of crude oil and leaded gasoline, and vehicle exhaust emissions [45]. Cr may come from metallurgy, electroplating, paper making, machinery manufacturing, and other industries, mainly used in metal surface treatment and printing and dyeing industries [46]. It can be inferred that Source 1 mainly represents the pollution of industrial sewage and air emissions from coal combustion.

According to the comparison of different soil layers in Figure 3, it can be found that the change rule of source percentage among As, Mn, and V is almost the same, and Figure 4 shows that the concentration of As, Mn, Ni, and V is low near the riverbank and high far from the river. According to this, it can be judged that As, Mn, Ni, and V are from another pollution source, and the pollution source is more evenly distributed in the slightly distant places on both sides of the river. It has been found that the element As is rare in the natural environment and is an important component of chemical fertilizers and pesticides. It is also a major pollutant in the cinder dump area [47]. The low-grade superphosphate and phosphate rock powder in chemical fertilizers contain trace As [48]. The heavy metal element Ni is mainly used in the electroplating industry or as a catalyst in the industry [46]. Mn is mainly used in the production of glass colorants, paints, pigments, matches, soaps, artificial rubber, plastics, pesticides, and other industries. In addition, there are a large number of Mn compounds in soil. V Pollution mainly comes from organic and inorganic chemistry, glass and ceramic manufacturing, textile, electronics, pigment, paint, leather, national defense, and other industries. Generally, exhaust gases pollute the atmosphere and can settle after diffusion. It can be concluded that source 2 mainly represents agricultural pollution and light industrial pollution emissions.

However, there is no single source of heavy metals. According to the Pearson correlation analysis, Ni and other heavy metals are not related; however, this does not mean that it is from another pollution source. Figure 4 shows that the concentration distribution of Ni is high in the south and low in the north, with a low concentration at the sampling point and a high concentration around the sampling point. This indicates that the Ni concentration is the result of the interaction between Sources 1 and 2, and there is no primary source connecting the two sources.

## 6. Conclusions

The sources of heavy metals in soils along the Shuimo River in Urumqi were analyzed using two models, PMF and UNMIX, and the results showed that the combination of the two methods improved the credibility of the source analysis, and the results of the analysis of these two models had strong similarity and could be verified with each other. Although the basic principles of the two models are different, similar conclusions can be drawn for the same set of data after simulation and analysis, which shows that the two models are fully applicable to this set of data. This reduces to a certain extent the possibility of errors in a single software run and further improves the credibility of the source analysis results. Combined with the ordinary kriging interpolation method, it can be concluded that the four heavy metals As, Mn, Ni, and V mainly originate from agricultural pollution and light industrial pollution emissions, and the four heavy metals Zn, Pb, Cr, and Cu mainly originate from industrial emissions of sewage and air pollution from coal combustion. However, a comparison of Figure 4 shows that some pollutants mainly from Source 2 have the same distribution characteristics as Source 1, such as Ni and V. This shows that the sources of many heavy metals are not single, and even for pollutants whose main concentration contribution comes from source 1, there is a significant part of the contribution from source 2, or even other sources outside the two sources, and vice versa. Therefore, to quantify the number of pollutants discharged from different emission enterprises, we need a large amount of information on soil heavy metal concentrations in a more refined and longer time dimension.

Combined with the statistical characteristics of soil heavy metal concentration, the average concentration of heavy metals in different soil layers is not significantly different, which may be due to the difficulty of downward migration of pollutants in hard soil locations where deep excavation is not possible and the concentration of pollutants is low, but due to the limitation of sampling tools we did not collect, while the downward migration of pollutants is faster in places where the soil is soft and easy to collect, resulting in the concentration between the upper and lower layers is not very different, these deep layers As soon as the data were aggregated, the concentration of pollutants in the deep layer was not very different from that in the surface layer, so the average values of pollutant concentrations in the three layers of soil samples were similar in the end.

The overall analysis concluded that the soil environmental conditions along the Shuimo River were generally good. However, as a big city with a population of more than 4 million, the situation around the river is likely to deteriorate further with the continuous influence of human activities. Therefore, to create a good living environment for the residents along the river, continuous monitoring of the soil heavy metal concentrations along the Shuimo River is needed. Subsequently, quantitative studies on the sources of heavy metal pollution can be conducted to analyze the sources of various pollutants in more detail, thus providing more feasible policy recommendations for river environmental protection and providing a scientific basis for subsequent studies related to the precise control of the sources of heavy metal pollutants in the soil along the river.

## Figures and Tables

**Table 1:** Statistical analysis results of heavy metals in soils along the Shuimo River.

Soil Depth (cm)	Heavy Metals	ω (mg/kg)							Variant Line Number (%)
		Minimum Value	Mean Value	Maximum Value	Median Value	Standard Deviation	BackGround Value	Filtered Value	
0–20	As	6.72	12.69	43.49	11.67	5.13	11.2	60	40.46
	Pb	10.32	20.60	66.49	18.59	8.21	19.4	800	39.87
	Zn	46.79	79.07	230.14	69.38	29.89	68.8	/	37.80
	Cu	18.09	39.01	144.41	37.69	12.85	26.7	18,000	32.95
	Ni	29.57	49.29	86.74	47.55	13.07	26.6	900	26.53
	Mn	377.08	529.00	769.47	525.68	66.05	688	/	12.49
	Cr	20.75	52.40	187.68	50.68	18.83	49.3	/	35.93
	V	70.24	112.31	175.77	112.66	15.24	74.9	/	13.57
20–40	As	6.06	12.65	41.61	11.80	4.92	11.2	60	38.92
	Pb	9.07	19.53	48.47	17.31	7.42	19.4	800	37.98
	Zn	43.65	78.94	264.57	66.62	38.23	68.8	/	48.43
	Cu	20.92	37.40	103.88	35.40	11.72	26.7	18,000	31.33
	Ni	30.61	49.74	92.49	46.77	12.91	26.6	900	25.95
	Mn	334.71	545.36	1413.36	531.02	117.17	688	/	21.49
	Cr	20.43	56.02	252.82	52.86	27.02	49.3	/	48.23
	V	58.57	112.63	182.32	114.03	15.95	74.9	/	14.16
40–60	As	5.58	12.89	45.64	11.71	5.61	11.2	60	43.57
	Pb	9.46	20.10	70.43	17.71	9.97	19.4	800	49.63
	Zn	22.64	76.98	323.71	66.53	36.04	68.8	/	46.82
	Cu	19.1	39.34	101.43	36.83	13.63	26.7	18,000	34.65
	Ni	30.44	50.97	83.81	49.67	12.38	26.6	900	24.29
	Mn	261.17	543.00	1307.35	532.55	116.88	688	/	21.53
	Cr	17.5	50.90	128.46	49.86	16.14	49.3	/	31.70
	V	44.38	111.74	181.42	112.35	18.50	74.9	/	16.55

Note: The background values of soil heavy metals were selected from the soil heavy metal concentration of layer a in the Xinjiang Uygur autonomous region divided by administrative provinces and cities (because the soil of layer B in arid areas is not fully developed, only layers a and C were selected in the sampling process. Layer a is the surface layer of soil, and layer C is the parent material of soil formation; therefore, layer a was selected).

**Table 2:** Pearson correlation coefficient of soil heavy metal contents at different sampling depths along the Shuimo River.

Sampling Depth (cm)	Heavy Metal	As	Pb	Zn	Cu	Ni	Mn	Cr
0–20	As	1.00						
	Pb	0.14	1.00					
	Zn	0.14	0.69 **	1.00				
	Cu	0.30 *	0.30 *	0.67 **	1.00			
	Ni	0.04	−0.08	−0.05	0.05	1.00		
	Mn	0.28	0.07	0.14	0.23	0.22	1.00	
	Cr	0.11	0.39 *	0.48 *	0.22	−0.17	0.19	1.00
	V	0.45 *	0.17	0.03	0.17	0.12	0.35 *	0.23
20–40	As	1.00						
	Pb	0.12	1.00					
	Zn	0.12	0.86 **	1.00				
	Cu	0.32 *	0.70 **	0.74 **	1.00			
	Ni	0.13	0.10	0.15	0.28	1.00		
	Mn	0.34 *	0.18	0.23	0.33 *	0.03	1.00	
	Cr	0.11	0.45 *	0.47 *	0.42 *	0.20	0.09	1.00
	V	0.34 *	0.21	0.14	0.27	0.14	0.29	0.31 *
40–60	As	1.00						
	Pb	0.22	1.00					
	Zn	0.12	0.75 **	1.00				
	Cu	0.44 *	0.59 *	0.64 **	1.00			
	Ni	0.25	0.21	−0.01	0.11	1.00		
	Mn	0.05	0.07	0.18	0.14	0.11	1.00	
	Cr	0.41 *	0.16	0.27	0.31 *	−0.01	0.05	1.00
	V	0.26	−0.01	−0.06	0.27	0.25	0.07	0.24

Note: ** indicates significant correlation at 0.01 level (bilateral), * indicates a significant correlation at the 0.05 level (bilateral).

## Data Availability

The data presented in this study are available on request from the corresponding author. The data are not publicly available due to the data involve sensitive information such as the environmental status of Urumqi.
